# Supplementary material for: Causal interplay between lactose intolerance and gut microbiota: a combined bidirectional Mendelian randomization and in vivo validation study
Source: Front Nutr. 2026 Jun 1;13:1803337. doi: 10.3389/fnut.2026.1803337 (PMC13265576; doi:10.3389/fnut.2026.1803337)
Supplement: Supplementary file 1 [file Data_Sheet_1.zip › supplementary materials/Forward/leave one out cross validation/ebi-a-GCST90027818.finngen_R12_E4_LACTONAS.pdf]

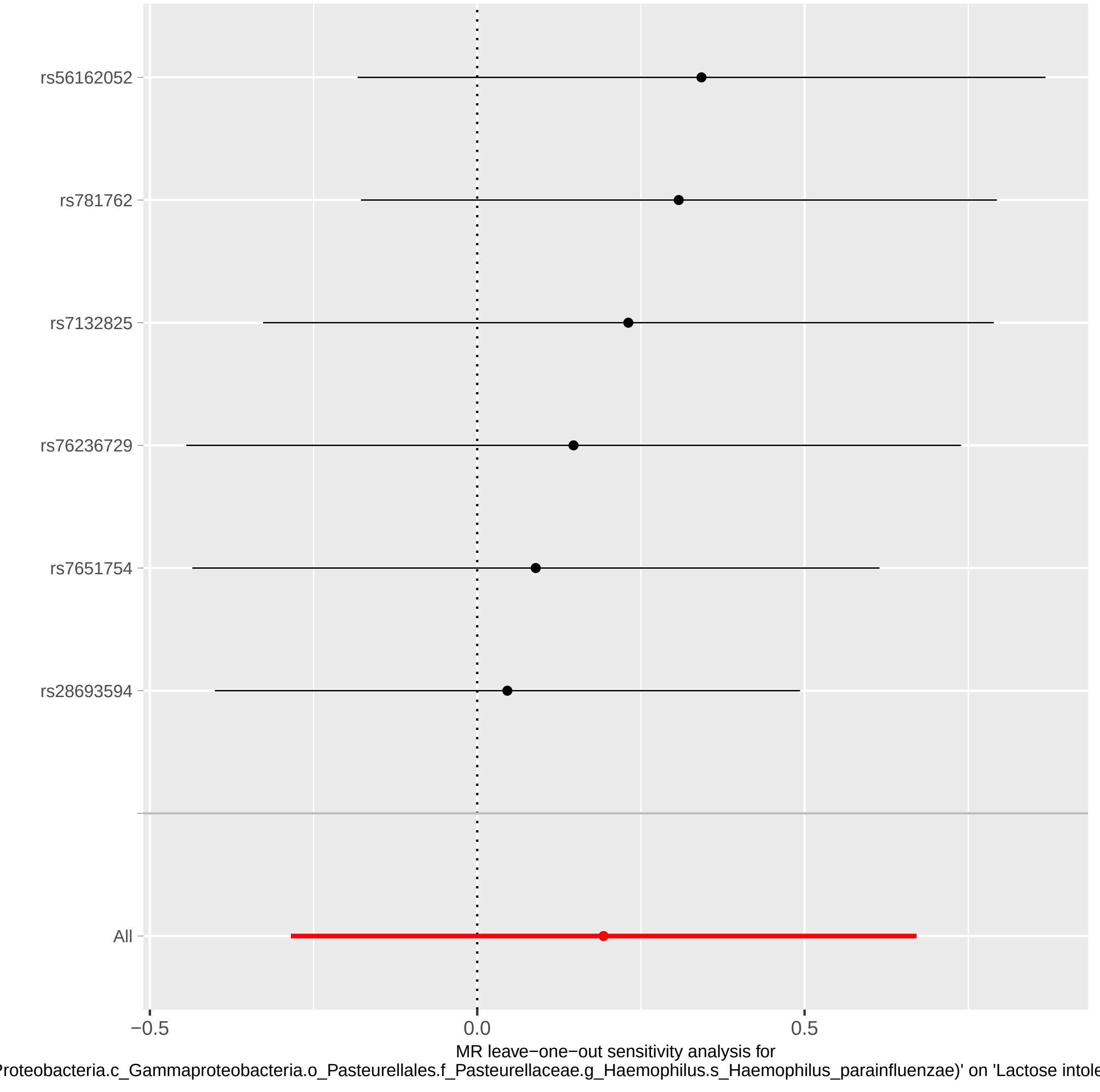
rs56162052
rs781762
rs7132825
rs76236729
rs7651754
rs28693594
All
−0.5
0.0
0.5
MR leave−one−out sensitivity analysis for
Proteobacteria.c_Gammaproteobacteria.o_Pasteurellales.f_Pasteurellaceae.g_Haemophilus.s_Haemophilus_parainfluenzae)' on 'Lactose intole
